# Supplementary material for: The Northumberland Exercise Referral Scheme as a Universal Community Weight Management Programme: A Mixed Methods Exploration of Outcomes, Expectations and Experiences across a Social Gradient
Source: Int J Environ Res Public Health. 2020 Jul 23;17(15):5297. doi: 10.3390/ijerph17155297 (PMC7432420; doi:10.3390/ijerph17155297)
Supplement: Supplementary file 1 [file ijerph-17-05297-s001.pdf]

International Journal of
Environmental Research and Public Health

MDPI

Supplementary file

The Northumberland Exercise Referral Scheme as a universal community weight management programme: a mixed methods exploration of outcomes, expectations and experiences across a social gradient.

Caroline J. Dodd-Reynolds[1,2,3*], Dimitris Vallis[3] Adetayo Kasim[2,3,4], Nasima Akhter[2,4] and Coral L. Hanson[5]

[1]Department of Sport and Exercise Sciences, Durham University, Durham, UK; caroline.dodd-reynolds@durham.ac.uk
[2]Wolfson Research Institute for Health and Wellbeing Physical Activity Special Interest Group, Durham University, Durham, UK.
[3]Durham Research Methods Centre, Durham University, UK; dimitris.vallis@durham.ac.uk
[4]Department of Anthropology, Durham University, UK; a.s.kasim@durham.ac.uk
nasima.akhter@durham.ac.uk
[5]School of Health and Social Care, Edinburgh Napier University, Edinburgh, UK; C.Hanson@napier.ac.uk
*Correspondence: caroline.dodd-reynolds@durham.ac.uk

Table S1. Patterns for missing data.

Pattern	Weight % (n)	BMI % (n)	Waist % (n)	Godin % (n)	Godin (HCS) % (n)
**OOO**	33.99%	33.77%	33.77%	14.32%	15.38%
**OOM**	12.04%	12.00%	11.71%	20.41%	19.46%
**OMO**	4.15%	4.26%	4.07%	1.36%	1.25%
**MOO**	0.48%	0.48%	0.48%	0.29%	0.26%
**OMM**	48.35%	48.24%	47.76%	58.48%	58.66%
**MOM**	0.18%	0.15%	0.33%	0.73%	0.66%
**MMO**	0.00%	0.04%	0.00%	0.00%	0.15%
**MMM**	0.81%	1.06%	1.87%	4.26%	4.19%

M: Missing; O: Observed. E.g. OOO Observed data for participant at all three timepoints; OOM, Missing data for participant only at timepoint 3.
